# A reflexive artificial intelligence governance for transformative change in sustainability

**DOI:** 10.1007/s13280-026-02405-y

**Published:** 2026-04-29

**Authors:** Anna Hausmann, Tuuli Toivonen, Gonzalo Cortés-Capano

**Affiliations:** 1 Department of Biological and Environmental Science, School of Resource Wisdom, University of Jyväskylä, Survontie 9c, 40500 Jyväskylä, Finland; 2 Faculty of Organic Agricultural Sciences, University of Kassel, Witzenhausen, Germany; 3 Digital Geography Lab, Department of Geosciences and Geography, Faculty of Science, University of Helsinki, Helsinki, Finland; 4 Helsinki Institute of Sustainability Science (HELSUS), University of Helsinki, 00014 Helsinki, Finland; 5 Department of Agricultural Economics and Rural Development, University of Göttingen, Göttingen, Germany

**Keywords:** Environmental governance, Environmental justice, Ethics, Reflexivity, Transformative change

## Abstract

Artificial intelligence (AI) is increasingly integrated in sustainability governance, yet most applications remain oriented towards optimisation and prediction, reducing complex social–ecological issues to technical problems. This narrow focus neglects plural values, lived experiences, and democratic judgement essential for transformative change. We advance a reflexive AI governance approach that treats AI as a socio-technical assemblage shaping problem framing, knowledge legitimisation, and authority distribution. Synthesising material, technical, epistemic, and ethico-political challenges, the paper draws on Aristotelian notions of *techne*, *episteme*, and *phronesis* to outline three reflexivity dimensions: design, epistemological, and engagement. Using a four-phase governance cycle and a protected area management scenario, we show how reflexivity can help align AI with plural, justice-oriented transformation pathways. Reflexive AI governance grounded in sustainability’s visions fosters deliberation, inclusivity, and ecological sufficiency, enabling democratic capacities over whether and how AI should be used, including the legitimate possibility of non-use, restriction, or withdrawal.

## Introduction

Humans and non-humans are facing an intertwined sustainability crisis, marked by climate change, biodiversity loss, and widening socio-economic inequalities, which undermines the resilience of Earth’s life support systems (Lawrence et al. 2024). Responding to this polycrisis requires systemic transformations in worldviews, institutions, and everyday practices, as emphasised by the recent Transformative Change Report from the Intergovernmental Platform for Biodiversity and Ecosystem Services (IPBES) (O’Brien et al. 2024). Artificial intelligence (AI), as a rapidly advancing socio-technical assemblage, is increasingly envisioned as a powerful catalyst for advancing sustainability goals (Nishant et al. 2020; Silvestro et al. 2022; Reynolds et al. 2025; Creutzig et al. 2026). In biodiversity conservation, for instance, AI tools have been promoted for delivering more rapid, cost-effective, and comprehensive insights into ecological processes, management decisions, and wildlife protection (Silvestro et al. 2022; Reynolds et al. 2025). However, critical scholarship highlights numerous problems and negative consequences implied in the design and use of AI in sustainability, ranging from environmental impacts to biased knowledge creation and ethical questions (Galaz et al. 2021; Coeckelbergh 2023; Schütze 2024; Büscher 2025; Raymond et al. 2025; Sandbrook 2025). Without deeply engaging with these tensions, even well-intentioned AI applications risk replicating the very conditions driving unsustainable practices (Madianou 2021; Kloppenburg et al. 2022).

AI encompasses diverse analytical and generative systems, such as machine learning and neural networks, designed to perform tasks that mimic human-like reasoning, such as solving problems, making predictions, communicating, or making autonomous decisions (Gil de Zúñiga et al. 2023). Analytical systems are trained to recognise, classify, or predict patterns in data by learning boundaries between categories (e.g. support vector machines, convolutional neural networks, and other supervised machine learning). Analytical AI has dominated environmental inquiries, supporting, for instance, species identification from camera trap images and conservation monitoring, mapping land-cover, deforestation and pollution, waste management, and enhancing precision agriculture (Ullah et al. 2025). Generative AI (GenAI), in turn, is designed to produce new content, such as text, image, and multimodal synthesis, creating new plausible samples based on learned patterns in existing data and labels (e.g. diffusion models, large language models like ChatGPT).^1^ In sustainability domains, GenAI is expanding the scope of AI’s influence by simulating alternative climate or land-use scenarios, generating educational materials and policy briefs, facilitating participatory approaches, visualising diverse futures, and offering recommendations for developing nature-based solutions (Richards et al. 2024; Raymond et al. 2025). In addition, emerging socially attuned AI systems (Humanised AI), which are designed to interpret emotions and interact with users, are beginning to influence sustainability practice by shaping how people engage with information, raising new opportunities for participation but also new risks for manipulation and dependency (Raymond et al. 2025). These developments position AI beyond being only an analytical tool, to become a powerful mediator of how sustainability futures are represented, narrated, and imagined.

Mainstream narratives of “AI for sustainability” often rest on the promise that digital intelligence can optimise decision-making and deliver better environmental performance (Francisco and Linnér, 2023). However, sustainability transformations are political, situated, and plural processes (Avelino et al. 2024), shaped by contestation over values, histories, power, and unevenly distributed vulnerabilities (O’Brien et al. 2024). For example, a renewable energy project may advance decarbonisation while simultaneously threatening cultural landscapes or local autonomy, or certain biodiversity conservation actions can improve ecological resilience yet restrict subsistence practices. Therefore, transformative change is not only about improving performance within existing systems, but about renegotiating what counts as success, whose knowledge and authority are recognised as legitimate, and which futures are considered desirable and rendered possible (Bennett et al. 2019; Loos et al. 2024). Although initiatives such as “AI for Good” and “Responsible AI” seek to align AI with accountability and justice to address societal goals and environmental sustainability (Greif et al. 2024), technologies are never neutral tools as they embed political visions of governance and social order (Jasanoff and Kim 2015). Framings of AI for sustainability often reproduce dominant discourses of ecological modernisation, which define it as a technical optimisation challenge (Francisco and Linnér, 2023). This reinforces techno-solutionist imaginaries, in which more data and smarter models are presumed to resolve complex socio-ecological crises, thereby obscuring the structural inequalities and contested values, which transformative change efforts aim to address (Nutas 2024; Schütze 2024). A reflexive engagement with AI’s epistemic, ethical, and political commitments is therefore imperative to ensure that its use enables rather than constrains just and plural sustainability transformations (Galaz et al. 2025).

Reflexivity refers to the examination of one’s assumptions, positionalities, and effects in shaping knowledge and action (Voss et al. 2006). It has long underpinned efforts in science and technology studies to contest the neutrality of science and the authority of technological expertise (Haraway 1988; Jasanoff 2004). In pragmatist philosophy, reflexivity informs abductive reasoning, iterative problem framing, and democratic inquiry (Popa et al. 2015; Cortés-Capano et al. 2025a). Within sustainability science, it is recognised as a cornerstone of co-production, ethical deliberation, and inclusive governance (Voss et al. 2006; Lazurko et al. 2025). Governance involves the structures, processes, and norms shaping collective action and decision-making, with polycentric and participatory approaches often advocated as normative ideals for addressing complex sustainability challenges (Patterson et al. 2017). Governance practices are inherently techno-political, shaped by actor networks, institutional arrangements, and infrastructures, and involve contestation over justice, progress, and ecological responsibility (Mouffe 1999; Scoones et al. 2020). Despite progress across ethical and responsible AI, reflexivity has remained narrow to procedural standards of participatory approaches, ethical guidelines and principles such as fairness, transparency, and explainability (e.g. lawful, ethical, and robust) (Galaz et al. 2021; Gianni et al. 2022). Such approaches tend to overlook the limits of inclusion, when participation incorporates marginalised actors without addressing underlying power structures (Liboiron 2021), and the limits of ethics, which often focus on improving systems by design, rather than questioning how AI shifts authority and influence (Kalluri 2020). As a result, ethics and inclusion risk becoming technical add-ons rather than engagements with the political and normative considerations of AI.

Building on current debates in the literature on challenges and responsibilities of AI use in sustainability (e.g. Falk and van Wynsberghe 2024; Nutas 2024; Galaz et al. 2025; Sandbrook 2025), this perspective paper aims to critically rethink AI’s role within sustainability transformations. Specifically, we (i) establish a normative–epistemic grounding for context-sensitive and ethically responsive AI governance and (ii) show how reflexivity can be operationalised to better support future use of AI for transformative change. To this aim, we advance a governance approach grounded in the Aristotelian notions of *techne* (technical know-how), *episteme* (knowledge production), and *phronesis* (practical wisdom). To introduce these dimensions, we first present an overview of the main material, technical, epistemic, and ethical–political challenges that shape how AI currently operates in sustainability contexts. To do this, we purposively reviewed academic literature from diverse fields, such as science and technology studies, sustainability science, environmental ethics, epistemology, and digital governance. Building on this diagnosis, we introduce the reflexive practice, which can guide engagement with how AI systems are designed, legitimised, and governed within complex socio-ecological–technical systems. Finally, to illustrate this approach, we present a narrative case, focusing on a multi-stakeholder governance scenario in the context of overtourism and protected areas management, aiming at demonstrating how diverse AI applications might be governed reflexively.

## Reflexive AI governance for transformation

AI systems, both analytical and generative, are increasingly mobilised to advance sustainability goals across the IPBES approaches for transformative change (Table 1). However, AI systems are developed and deployed within specific ontological (i.e. how realities are defined and enacted) and epistemological (i.e. what is recognised as valid knowledge) commitments, which give rise to technical, epistemological, and ethical–political challenges in sustainability governance (Coeckelbergh 2023; Nutas 2024; Schütze 2024; Sandbrook 2025). The use of AI without explicitly engaging with these challenges may inadvertently shape which knowledge systems are encoded, which infrastructural and innovation pathways are scaled, and whose interests and values are prioritised in sustainability governance (Table 1).

**Table 1:** Challenges of AI across the six IPBES approaches to transformative change from O’Brien et al. (2024)

IPBES approach to transformation	Examples of AI uses	Main assumptions	Risks for transformations
**System change** (transforming visions, objectives, feedbacks, system structures)	Model system dynamics, detect early warnings, automate monitoring, and simulate alternative futures	Reductionist and predictive epistemologies. Complex systems can be captured and predicted through data and models	Ignoring uncertainty, emotions, and relational dimensions; narrowing transformative imagination; reinforcing hierarchical control and linear thinking
**Structural change** (reconfiguring governance, institutions, and power)	Evaluate policy outcomes, detect inequities, and support regulatory design	Systemic problems can be solved through optimisation and computational reconfiguration	Algorithmic efficiency can substitute political deliberation, shifting authority from citizens to technical systems
**Inner transformation** (shifting values, beliefs, and relationality)	Analyse discourse, emotions, and values; generate narratives and nudges for behavioural change	Values and emotions can be modelled, measured, and steered computationally	Transformation as behavioural engineering: reducing care, emotion, and spirituality to quantifiable data
**Empowerment** (fostering agency and recognition of marginalised actors)	Facilitate participatory approaches, translation, and visibility of local voices	Legitimacy lies in quantifiable or expert-validated knowledge; participation can be automated	Algorithmic bias and data inequalities exclude or co-opt communities; reproducing epistemic injustice; weakening grassroots autonomy, depoliticising participation
**Knowledge co-creation** (weaving plural knowledges and co-designing futures)	Integrate diverse data (scientific, local, Indigenous); generate shared maps and scenarios	Different kinds of knowledge can be standardised and integrated through hierarchical data synthesis	Reducing epistemic diversity by tokenising non-scientific knowledge; turning co-creation into instrumental hierarchical validation and integration
**Science and technology** (aligning innovation with inclusivity and ethics)	Detect bias, integrate practical knowledge, co-design responsible and sustainable AI	Nature and society can be governed computationally; technological innovation ensures progress	Reinforces extractivist and efficiency logics; risks technological lock-in and depoliticisation of systemic sustainability issues

**Fig. 1:**
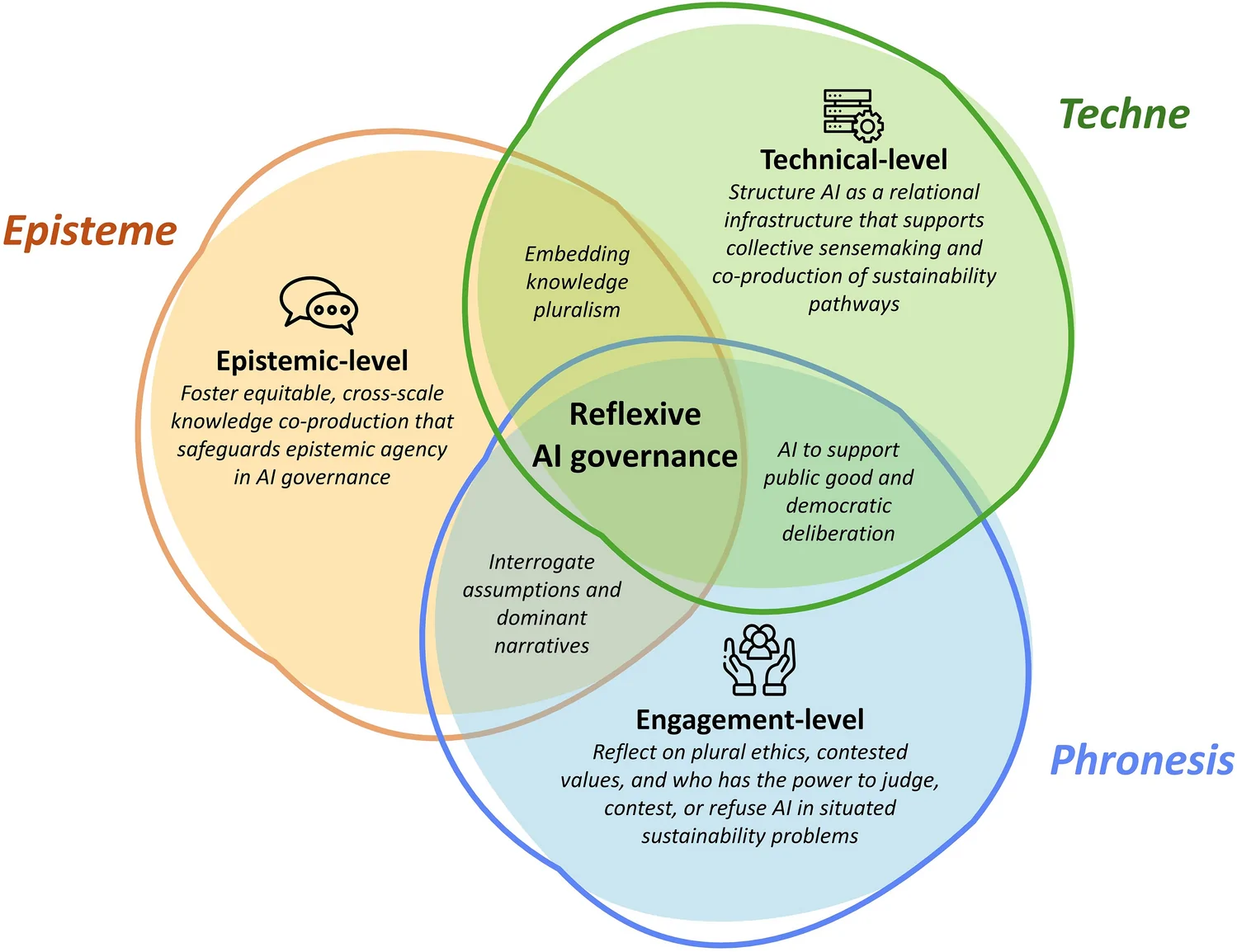
Integrating *techne*, *episteme*, and *phronesis* spheres into technical, epistemic, and engagement-level reflexivity in AI governance

A reflexive AI governance challenges solutionist or technocratic tendencies and aims at anchoring AI design, deployment, and use on critical reflection over assumptions, values, materialities, and power dynamics. To do so, we approach AI not as a neutral tool but as an embedded, co-evolving socio-technical actor within sustainability transformations. In order to cover different dimensions, we argue that reflexive AI governance should focus on three interrelated spheres of reflexivity (Fig. 1, Table 2): (1) a design-level reflexivity (*techne*) that attends to the practical crafting of AI systems, focusing on materiality, infrastructure, care, and stewardship to promote inclusivity, transparency, adaptability, and responsiveness; (2) an epistemic-level reflexivity (*episteme*) that interrogates the knowledge systems and assumptions underpinning AI, including how data, models, uncertainty, and partiality shape sustainability problem framings, and (3) an engagement-level reflexivity (*phronesis*), that addresses the ethical and political dimensions of AI governance, focusing on power, agency, democratic contestation, and collective judgement over when, how, and whether AI should be used.

**Table 2:** Key issues and guiding questions for reflexive AI governance according to *techne*, *episteme* and *phronesis*

Dimension	Reflexive issue	Guiding questions	Aspiration
*Techne*	The how of AI: reflecting on the role of optimisation algorithms and their social and ecological footprint	How can AI be built and maintained responsibly, accounting for social and ecological costs?	Reorient AI design from optimisation to care, embedding ecological limits and social reciprocity into code, infrastructure, and metrics, treating maintenance, repair, and de-growth as technical virtues
*Episteme*	The what/by whom/for whom of AI: what data and knowledge systems encode, who produces it, and for whom it is intended. Is AI all-knowing?	Whose knowledge and realities are represented or excluded in AI systems?	Cultivate pluriversal AI ecologies that interlink algorithmic reasoning with Indigenous, local, and experiential knowledge, transforming data infrastructures into spaces of epistemic dialogue rather than extraction
*Phronesis*	The why of AI: questioning AI as an objective solver of problems in light of ethical and political implications	Should AI be used in this context, for whom, and towards what kinds of futures? Who benefits and who holds power within the AI infrastructure, and who is excluded? Are the resulting pathways socially just and ecologically meaningful? And, in light of these considerations, what constitutes the wisest course of action?	Institutionalise collective wisdom in AI governance, where communities co-decide whether, how, and why AI should intervene, treating contestation, restraint, or non-use as legitimate and ethical outcomes

### *Techne*: Design-level reflexivity on material–infrastructural challenges

The IPBES science and technology pathway envisions transformative change emerging through the widespread social adoption and scaling of innovation, where the scientific and technical advances are intended to unlock new sustainability trajectories (Table 1). However, the transformative potential of innovation cannot be assumed independently of the material and institutional conditions under which it is produced and governed (Kloppenburg et al. 2022; Wickberg et al. 2024). A central challenge of AI for sustainability lies in AI’s own unsustainable material footprint (Falk and van Wynsberghe 2024). Despite efforts to align AI development with low-carbon transitions (Stern et al. 2025), its infrastructures remain resource-intensive and environmentally damaging (Wang et al. 2024). Data centres are already contributing to 1–1.4% of global CO_2_ emissions, an amount expected to rise as companies expand facilities with renewed investments in fossil fuel infrastructures (Jha et al. 2025). The rapid expansion of AI products and services has also escalated the demand and consumption of freshwater for server cooling and electricity generation (Li et al. 2025).

These environmental burdens are part of broader socio-ecological impacts driven by extractive economic logics, ranging from data capitalism to energy and labour-intensive production chains (Crawford^ 2021^; Schütze 2024). AI hardware depends on mineral supply chains involving graphite, lithium, and boron, whose extraction generates groundwater depletion, contamination, greenhouse gas emissions, and socio-ecological conflicts (Wang et al. 2024). These supply chains often exacerbate exploitative labour conditions and reinforce global inequalities, disproportionately affecting regions of the Global South (Regilme 2024). In this context, scaling AI risks further extending extractivist and neocolonial relations, even when mobilised under the guise of transformative change (Madianou 2021; Regilme 2024).

Design-level reflexivity (*techne*) focuses on material and infrastructural challenges by scrutinising how AI systems are designed, optimised, and sustained in practice. *Techne* concerns the craft and material practice, and institutional embedding of AI systems, interrogating what forms of optimisation they privilege and at what social and ecological costs (Table 2). This involves the scrutiny of the full life cycle of AI from data sourcing and model training, to energy use, mineral extraction, labour conditions, and e-waste, in relation to maintenance, repair, and durability as technical virtues and design principles (Kerschner et al. 2018). This perspective builds on existing efforts, such as “ethics-by-design” (Brey and Dainow 2024) and multi-source data integration (Bawack et al. 2025), by embedding social–ecological care and responsibility directly into system architectures, infrastructures, supply chains, and data governance. Instead of scaling innovation *per se*, design-level reflexivity shifts the focus towards transforming the material and data infrastructures that enable it, thereby situating AI within ecological limits as a relational, rather than extractive, technology. However, while a large part of the attention has been given to design-level improvements, this alone cannot address the deeper epistemic and political questions, and reflexivity in *techne* must be complemented by scrutiny of whose knowledge is encoded and how these affect ecological, social, and political outcomes.

### *Episteme*: Epistemic-level reflexivity on knowledge challenges

AI systems increasingly mediate sustainability transformations by identifying patterns, recommending interventions, and informing decisions across different IPBES transformative approaches (Table 1). While often presented as objective or “evidence-based”, their outputs are shaped by architectures that collapse uncertainty into definitive predictions, producing confident-sounding recommendations, which give an impression of certainty and simplicity that mask ambiguity and contestation (Shojaee et al. 2025). In conversational systems, these dynamics are further amplified by design incentives to always provide an answer regardless of accuracy, truth, epistemic grounding, or positionality, going from occasional “hallucinating” to systemic “bullshitting” (Hicks et al. 2024). Even advanced Large Reasoning Models, designed to perform complex reasoning and real-world problem-solving (e.g. through Chain-of-Thought), remain constrained by these architectures, giving a false impression of rationality (Shojaee et al. 2025). This simplification limits AI’s capacity to engage with “wicked” sustainability problems, such as climate-energy trade-offs, ecosystem degradation, or food security, that involve multiple stakeholders, value conflicts, uncertainty, and nonlinear dynamics.

These limitations are not simply technical but arise from the epistemic infrastructures underpinning AI. AI systems are constituted by longstanding practices of “datafication” (van Dijck 2014), in which complex social and ecological phenomena are translated into abstracted quantifiable variables (e.g. carbon reduction or biomass increase). Such practices have long shaped sustainability science and environmental governance, for example, through the development of indicators intended to “measure what matters” in terms of well-being, ecosystem services, and natural capital (Costanza 2001). However, instead of simply representing realities, indicators and valuation techniques actively construct normative orders and policy priorities (Spash 2008). AI intensifies these processes as choices about what matters, whose knowledge is considered, and which futures are desirable are reflected in digital data (Crawford et al. 2014; Kitchin 2014). By privileging data-rich, standardised and widely represented knowledge, AI systems filter out marginalised or underrepresented perspectives, and through recursive use and training on its own narrow information diversity, can lead to “knowledge collapse”, constraining innovation and eroding cultural diversity (Peterson 2025). At the same time, corporate-driven constant demand for large-scale training data incentivises industrial data extraction from ecosystems, individuals, and communities, further collapsing complex socio-ecological relations into commodifiable metrics (Thatcher et al. 2016). As these epistemic selections are encoded into training datasets, optimisation criteria, and output formats, AI systems can shape what can be known and what becomes actionable (Ahmad et al. 2026). In this sense, overreliance on AI systems risks reinforcing managerial and efficiency-oriented logics, constraining the integration of plural epistemologies that are central to IPBES transformative approaches (Table 1).

AI can further narrow the space for diverse sources of expertise and perspectives to shape sustainability solutions (Urzedo et al. 2024). For example, AI chatbots like ChatGPT tend to privilege US-based male academic expertise and technocratic restoration framings (planting and reforestation techniques), neglecting non-tree species and ecosystems, environmental justice concerns, and holistic socio-ecological approaches (Urzedo et al. 2024). As training data are shaped by historical, often colonial legacies (Cieslik and Margócsy 2022), AI systems reproduce decision support frameworks that disproportionately encode Western experiences, epistemologies, and dominant cultural imaginaries (Mohamed et al. 2020). Within platform-capitalist environments, such visibility becomes a proxy for credibility and “truth”, concentrating epistemic authority in technology corporations (Hicks et al. 2024; Büscher 2025). For instance, by reproducing Western romanticised notions of “nature”, AI systems can shape collective sensemaking about what should be conserved and how (Luque-Ayala et al. 2024; Sandbrook 2025). Attention-maximising algorithms amplify viral, spectacular, or aesthetically pleasing representations of nature (e.g. charismatic species), while sidelining less spectacular, everyday places and practices (Büscher 2020). GenAI reproduces these patterns, reinforcing narrow rewilding aesthetics that render certain species, landscapes, and people absent from imagined futures (Wartmann and Cary 2026). As a result, rather than enabling plural, reflexive transformations, corporate platforms can shape conservation and sustainability agendas by narrowing epistemic diversity and re-inscribing hierarchical authority over what can be known, valued, and transformed (Büscher 2020).

Reflexive *episteme* helps address these challenges by interrogating which knowledge and perspectives AI encodes and legitimises (Table 2). Since AI is explicitly designed to perform epistemic functions (e.g. by processing, transforming, and generating knowledge), its legitimacy cannot rely on practical performance alone, but must instead be evaluated against context-sensitive epistemic standards, including how well it supports reliable inquiry, actionable understanding, and shared problem-solving in situated contexts (Alvarado 2023). Reflexive *episteme,* thus, entails questioning not only the data used to train AI systems, but also how models reason with data in terms of how validity, causality, and evidence are constructed. This approach treats knowledge as partial, situated, and entangled with power (Haraway 1988), promoting plural knowledges, decolonial reclamations of marginalised epistemologies, aligning AI governance with transformative, justice-oriented action (Lugonzo 2025; Prabakaran 2025; Arora et al. 2026).

To enact a reflexive *episteme*, institutionalised spaces can bring together domain experts, AI developers, and stakeholders affected by AI-supported data extraction and decision-making, to scrutinise model assumptions, abstractions, and causal logics and to configure systems so that uncertainty, disagreement, and partiality remain visible (Sardelli 2022; Coeckelbergh 2023). Together with reflexive *techne*, *episteme* fosters designers and engineers to build open, participatory infrastructures, enabling AI to support collective problem-solving processes (Gianni et al. 2022; Wilson 2022). These spaces should also include those whose behaviour is shaped by AI guidance (e.g. users interacting with recommendation systems), enabling them to question how their data are used, and what trade-offs are implied in algorithmic advice. These spaces enable meaningful involvement in how knowledge is produced, interpreted, and authorised, without requiring all actors to become model developers. Through abductive reasoning, stakeholders can explore alternative interpretations, pose new questions that matter to them, and sustain dialogue and contestation across plural knowledge systems (Cortés-Capano et al. 2025a, b). This contributes to safeguarding epistemic agency in terms of individuals’ and groups’ capacity to actively form, revise, and control knowledge amid AI mediation, to enable context-sensitive action crucial in transformative change (Caniglia et al. 2021). In doing so, it helps shift innovation logics from corporate-driven acceleration towards co-production, enabling diverse actors to shape both the purpose and infrastructures of AI systems.

Combined with design-level (*techne*), epistemic-level (*episteme*) reflexivity redirects AI to act as a facilitator of broader processes of transformation. However, as sustainability transformations are inherently normative and contested, shaped by plural problem framings and uneven power relations (Cortés-Capano et al. 2022), these reflections must be complemented by sustained attention to AI’s ethical and political dimensions.

### *Phronesis*: Engagement-level reflexivity on ethical and political challenges

By framing sustainability challenges as computational problems, AI risks depoliticising governance and shifting attention away from structural inequalities that underpin sustainability problems. AI systems, like chatbots, favour incremental, experience-based solutions to environmental challenges, avoiding systemic change, and downplaying accountability and links to broader social justice concerns (van der Ven et al. 2024). When plural and incommensurable values are conflated into standardised indicators, disagreements over meaning, justice, and responsibility are obscured and reframed as technical problems of optimisation (Martinez-Alier et al. 1998; Turnhout et al. 2014). Even when AI systems are designed to process heterogeneous and high-dimensional data, they ultimately rely on transforming this heterogeneity into commensurate metrics, so it can be compared and ranked. Normative trade-offs and value judgement are integrated in training data selection, target variables definitions, and the calibration of thresholds and weights that determine acceptable outcomes. These design choices encode assumptions about priority and substitutability that are often opaque, limiting stakeholders’ capacity to scrutinise, contest, or renegotiate how values are assessed and whose interests are prioritised.

For example, AI and remote sensing are increasingly used to optimise carbon market investments by relying on quantifiable metrics such as tree cover, carbon sequestration potential, and land-use data (Ferronato et al. 2025). However, these standardised indicators embed implicit value judgements that can obscure assumptions about data selection, model design, and prioritisation of trade-offs between ecological, social, and cultural objectives. These value-laden choices can shape whose interests are served and which outcomes are considered acceptable in context, overlooking that such decisions require situated practical judgement rather than purely technical optimisation. In such contexts, decision-making authority may become concentrated in Global North corporations or NGOs, potentially marginalising local and Indigenous governance and reinforcing historical patterns of political exclusion (Galaz et al. 2021; Redvers et al. 2025; Sandbrook 2025). The lack of transparency of AI systems further limits stakeholders’ ability to understand, question, or contest how outcomes are produced, and priorities balanced, raising concerns about communities’ capacity to scrutinise, challenge, or refuse AI-mediated interventions, which are core democratic principles of equitable sustainability governance (Coeckelbergh 2023, 2024; Patterson et al. 2024).

Because AI is built on technocratic and historically dominant models of sustainability, and trained on past data, it risks reproducing the very structures and practices that failed to address the underlying causes of the crisis, thereby hindering transformative change (van der Ven et al. 2024). This dynamic becomes particularly visible when models developed in data-rich industrial contexts (e.g. large-scale farming in the Global North) are applied to data-poor regions in the Global South (Galaz et al. 2021). For example, AI tools for precision agriculture trained on datasets from industrial farming and expertise from the Global North, include spatial and cultural biases that neglect agroecological or Indigenous practices (Urzedo et al. 2024). By optimising for yield, growth, or carbon metrics, these systems could prioritise fast-growing, high-biomass species irrespective of local ecological conditions, promoting monocultures in fragile or biodiverse ecosystems (e.g. exotic eucalyptus plantations on grassland biomes; Rocha et al. 2020). In doing so, AI can further entrench productivist logics and extractive models of production, contributing to landscape homogenisation, exacerbating inequalities and biodiversity loss (Cortés-Capano et al. 2025b).

AI systems increasingly influence public decision-making by mediating what information is seen in digital environments, how it is filtered and how choices are presented, challenging people’s ability to think, deliberate, and make their own decisions (Bolton et al. 2021). Through profiling, preference inference, and algorithmic ranking, AI-powered recommendation can steer users towards predefined options, narrowing how individuals perceive and evaluate their choices, and constraining deliberation and contestation (Coeckelbergh 2023). In this way, AI-driven nudging risks reducing citizens to passive recipients of prescriptive solutions, undermining individual and collective self-determination, and threatening the epistemic agency central to democratic life (Coeckelbergh 2023). These dynamics are reinforced by increasingly concentrated political economy of AI, where control over data, computational infrastructure, and model development is held by a small number of corporate actors in the Global North, shaping whose knowledge counts and whose interests prevail (Madianou 2021; Büscher 2025). These ethical and political challenges directly intersect with IPBES transformative concerns about empowerment, inner transformation, and structural change, where agency, authority, and participation are critical (Table 1).

*Phronesis*, or practical wisdom, provides a vital reflexive lens for AI governance in sustainability transformations, as the ability to discern the right course of action in specific, complex situations, balancing moral principles with contextual demands. While *techne* helps interrogate “how” AI is built, and *episteme* addresses “what” knowledge is encoded, “by whom” it is produced, and “for whom” it is intended, *phronesis* asks “why” AI should be used (Table 2), introducing the crucial dimensions of judgement, ethical reasoning, and contextual sensitivity into AI governance. AI’s contribution to sustainability transformations depends on whether actors retain the authority to interrogate, override, or refuse its processes and outputs (Bolton et al. 2021). Engagement-level reflexivity operationalises *phronesis* by mandating deliberation among those whose lives, values, and environments affect or is affected by AI-supported decisions. Instead of pre-empting ethical reasoning or providing resolutions to ethical dilemmas with static “in-design” solutions, *phronesis* allows stakeholders to exercise collective sensemaking around the role and impact of AI in transformation pathways. A set of questions (Table 2, Flyvbjerg 2008) may guide reflexive *phronesis* towards issues of future-making, power, and justice, when scrutinising AI in terms of human judgement, responsibility, and collective agency (Gianni et al. 2022; Coeckelbergh 2024).

Addressing such practices in the age of AI requires that democratic values are incorporated into the design, deployment, and evolution of socio‐technical systems themselves (Ohren et al. 2026). *Phronesis,* thus, calls for institutional transformations that safeguard democratic responsibility over efficiency gains, requiring new governance frameworks that embed ethical oversight and citizen participation (Zuiderwijk et al. 2021). Decentralising authority and shifting control from private corporations to public and community-led arrangements may promote governance grounded in algorithmic accountability and enhanced civic engagement. In this sense, AI's core functions, like data processing, modelling, and predictive analytics, can be treated as shared commons, collectively governed to support plural societal needs and sustainability transformations, rather than profit maximisation*.* For instance, through multi-actor forums, affected communities can critically assess and challenge whether AI deployments advance collective and societal well-being or instead reinforce commercial priorities at the expense of public good (Cohen and Suzor 2024). Moreover, democratic innovations, such as institutional spaces for agonistic contestation (Asenbaum 2025), citizen assemblies, and minipublics, might allow for extending discussions beyond fairness and transparency to affirm rights to restrain or oppose AI applications that reinforce corporate power concentrations (Cheng 2024). Public AI initiatives that emphasise democratic oversight, open-source modularity, cooperative models (Terras et al. 2025), and trusted data intermediaries can further reduce dependency on corporate monopoly, while organised workers and community-led governance models can help ensure AI advances equitable resource allocation and safeguards public sovereignty (Wickberg et al. 2024).

## A reflexive AI governance in protected area tourism

**Fig. 2:**
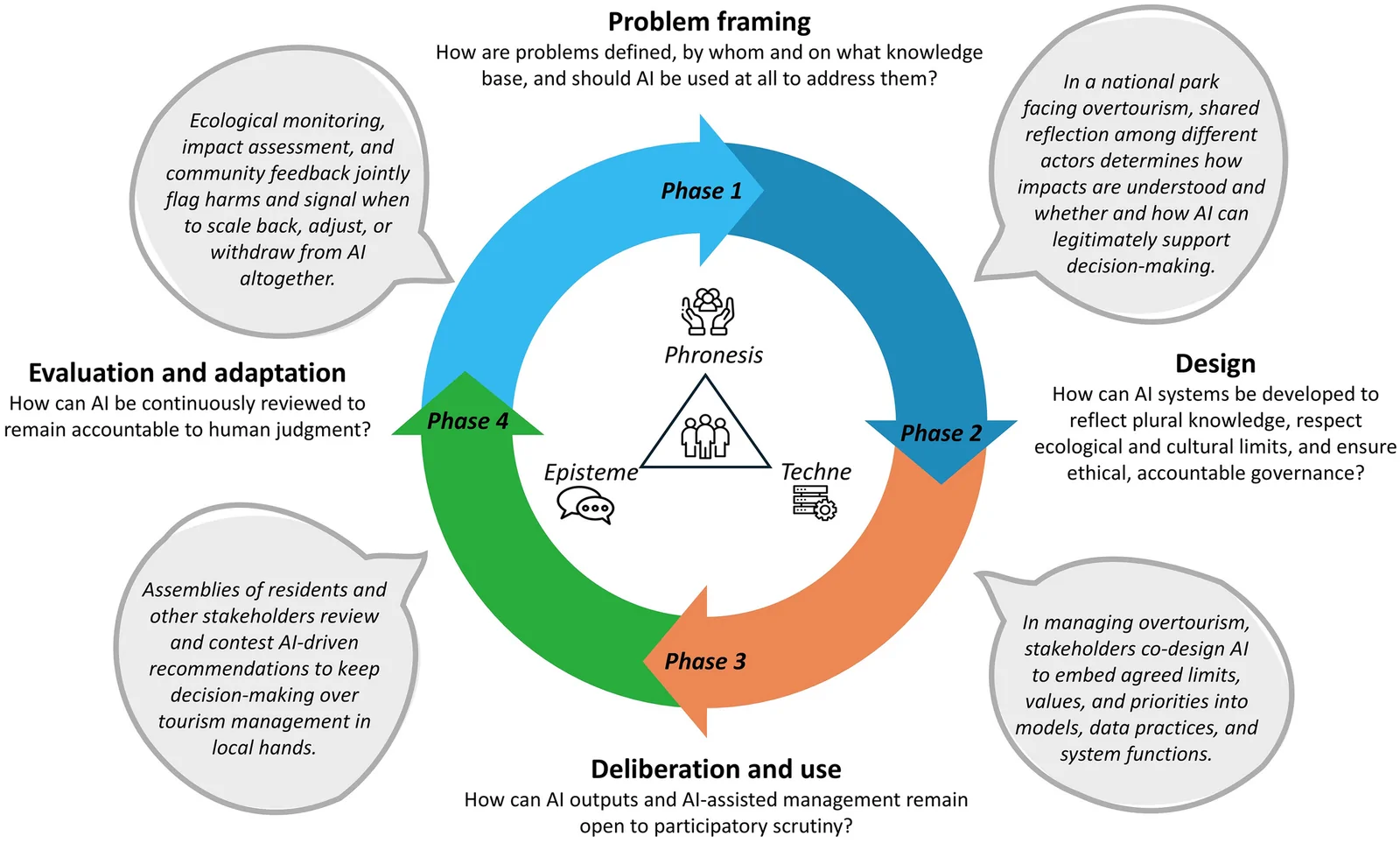
Reflexive governance cycle for AI, illustrating four phases to enhance the ethical, adaptive, and democratic capacity of AI in sustainability transformations. Each phase is paired with guiding questions and example responses from an overtourism management scenario involving diverse actors in protected areas. Practical wisdom (*phronesis*), technical practice (*techne*), and plural knowledge (*episteme*) are at the core of the cycle and embedded in each phase’s reflexivity practice

To provide a practice-oriented example of reflexive AI governance, we develop an illustrative narrative grounded in existing literature at the intersection of tourism, conservation, and digital technologies. We construct a scenario of management in a protected area, where various actors deploy AI applications and decision support systems to diagnose, frame, and address sustainability pressures from overtourism (Patrichi 2024). Overtourism is the rapid growth of visitors that exceeds multiple ecological, economic, social, and cultural capacities (Milano et al. 2019). Its management is characterised by complex, multi-actor coordination, value contestation, and uncertainty, making it a complex sustainability problem (Mihalic 2020). The following example does not describe a real empirical case, but it is built on relevant literature to demonstrate how reflexive AI governance could be implemented if such a case existed in practice. Reflexivity in this illustrative case is enacted through how actors interrogate AI systems across dimensions of *techne*, *episteme*, and *phronesis* (Table 2). They do so across four stages of AI integration: problem framing, design, deliberation and use, and evaluation and adaptation (Fig. 2). Residents, rangers, managers, tourism operators and visitors, engage as co-designers across different phases, involving mechanisms of public engagement (Wilson 2022), such as citizen assemblies, participatory monitoring boards, and co-management platforms.

### Problem framing

The illustrative case is set within a national park facing unprecedented crowds driven by viral social media attention (Solanky and Gupta 2021) and bringing economic, social, and physical disruptions to from trails, infrastructures, and landscapes to residents’ well-being (Milano et al. 2019). Local groups, conservation organisations, park managers, and tourism companies are turning to AI-powered decision support systems to help manage these overlapping pressures. Conservation scientists use AI facial recognition and acoustic sensors to detect human disturbance on wildlife from camera traps (Tuia et al. 2022) and to inform monitoring priorities, enforcement, or zoning decisions (Ojija et al. 2025). Park managers frame overtourism as a carrying-capacity problem and use AI predictive analytics to optimise visitor flows (Höpken et al. 2021). Tourism agencies use AI-powered personalised recommendation systems on a behavioural nudging strategy that redirect visitors towards less crowded or “eco-friendly” alternatives (Sansone et al. 2024).

These systems render overtourism legible primarily through patterns of movement, consumption, and congestion, which are dimensions readily measurable and actionable within AI logics. Yet they can overlook how communities and visitors themselves actually experience overtourism, especially issues of justice, autonomy, cultural meaning, and care for place (Rogowski 2025). Without reflexive governance, uncoordinated AI use risks concentrating power in corporate platforms, subordinating communities and visitors needs to commercial interests, reducing overtourism management to data-driven optimisation, or generating overdependence on AI systems that displace local judgement over acceptable impacts and desired futures.

In this example case, a reflexive approach creates spaces where AI is scrutinised against multiple problem framings. Through multi-actor workshops, storytelling sessions, and participatory mapping, protected area managers, researchers, tourism actors, and local communities can articulate and negotiate diverse framings of what counts as “disturbance” and whether, how, and to what extent AI can meaningfully support decision-making. Epistemic reflexivity is enacted as participants critically examine which knowledge (e.g. scientific monitoring data, local observations, historical uses) should inform AI systems to address different problem definitions. Through phronetic reflexivity, participants collectively confront normative questions AI cannot resolve, including how to balance ecological impact with local livelihoods, which culturally significant sites warrant priority, and how to ensure that AI-driven tourism management does not displace residents or restrict their access to public space in favour of visitors. *Techne*-oriented reflexivity allows asking how tools should be built and bounded so that practical skill serves scientific understanding and collective judgement in ways that addresses distributive and spatial justice, while considering appropriateness of AI’s use in terms of ecological footprint.

### Design

Normative constraints are translated into technical and governance design choices, ensuring that *techne*, *episteme*, and *phronesis* guide the design of systems architecture. By enacting *techne*-oriented reflexivity, park managers, conservation scientists, and technology developers make explicit choices about what AI systems are, and are not, built to do. Rather than defaulting to continuous real-time space–time tracking of individuals, systems are designed for periodic, site-specific and informed monitoring of visitors within existing ranger patrol infrastructure (e.g. camera trap settings) (Guidosse et al. 2025), or for anonymous crowd-detection systems (Santos et al. 2025), reducing energy demands and surveillance concerns. Models are selected for their maintainability and compatibility with local institutional capacities, enabling actors to manage and adapt AI systems independently while reducing dependency on external corporate platforms and mitigating risks of lock-ins, data privacy concerns, and loss of control over critical infrastructure. Data collection protocols are required to lowering resolution in sensitive or culturally significant sites and restricting collection that could expose indigenous knowledge without community consent.

Epistemic reflexivity at the design stage means specifying required knowledge sources upfront to ensure AI systems support robust, long-term conservation outcomes. A carrying-capacity model built solely on visitors counts and vegetation stress indices would render invisible the cumulative social and cultural pressures that residents experience, such as loss of access to significant sites, erosion of subsistence practices, displacement by visitor-oriented infrastructure. To prevent this, human in/on the loop (HITL) architectures (Monarch 2021) are used to integrate ranger observational records, historical land-use data, real-time sensor outputs, and visitor behaviour and experience insights (Chisom et al. 2024). Interface annotation allows non-technical users to contribute to contextual knowledge and flag anomalies, ensuring scientific understanding remains grounded in diverse, place-based evidence.

Phronetic reflexivity helps establishing the governance infrastructure that keeps technical and scientific choices accountable to collective, often plural values. This includes binding data stewardship agreements specifying ownership, permitted uses, and community withdrawal rights over data, written before any platform agreement is signed (Pritchard et al. 2022). To institutionalise cooperation in AI oversight, an independent ethics panel with conservation, community, legal, and tourism representatives, is constituted and mandated to review AI applications prior to deployment and at regular intervals.

### Deliberation and use

As AI systems move from design to deployment, the integration of *techne*, *episteme*, and *phronesis* reflexivity shifts from architectures to coordinated practices. Conservation scientists train supervised models on camera trap and citizen science data (e.g. iNaturalist or eBird), working with local knowledge holders to incorporate their situated knowledge and understandings into datasets and outputs (Mohamed et al. 2020). Park managers prioritise ecological regeneration, cultural significance, and community well-being over revenue or visitor satisfaction. Tourism operators adjust recommendation systems and digital storytelling towards regenerative experiences grounded in local relationships with places.

*Techne*-level reflexivity in this stage means deploying adjustable AI systems with built-in feedback channels, allowing models to be recalibrated as ecological or social conditions change (Monarch 2021). In practice, this might mean retraining models to account for seasonal dynamics or adjusting wildlife disturbance threshold following ranger observations, so that practical tools remain responsive to context. Epistemic-level reflexivity is enacted through ongoing engagement among diverse actors over which knowledge inform AI predictions, opening space to question and rebalance the privileging of sensor-based metrics over local, experiential, or local knowledge. Through phronetic reflexivity, actors collectively deliberate on ethical and normative tensions in use, including when AI should be adapted, limited, or not used where it conflicts with justice or cultural values.

### Evaluation and adaptation

AI-powered decision support is integrated within a structured, iterative assessment practice, covering system performance, social and ecological effects, and institutional dependencies. Evaluation moves beyond one-off assessment or validation towards ongoing scrutiny of intended outcomes, unintended consequences and distributional effects, asking not only whether AI works, but also whether its integration remains sustainable, just, and appropriately bounded over time.

Through reflexive *techne,* assessment examines forecasting accuracy, data quality, resource consumption, ecological footprint, and whether optimisation targets generate secondary pressures elsewhere. For instance, whether redistributing visitor flows inadvertently intensify use in ecologically fragile or culturally significant areas. Epistemic reflexivity helps actors revising which indicators define “success”, which experiences are excluded, and how model outputs reshape what decision-makers perceive as actionable problems. Together with *techne*, epistemic reflexivity also monitors signs of institutional overdependence and whether managers retain the ability to critically select, analyse, and interpret information generated or filtered by AI systems as well as the confidence to make decisions without it. Through phronetic reflexivity actors ask who benefits from AI-driven efficiencies, who bears risks, and how value is distributed (Buhmann and Fieseler 2021). For example, whether visitor redistribution revenues flow to local communities or external operators, and whether data stored on corporate infrastructure threaten data sovereignty. Where evidence shows inequitable outcomes or unacceptable ecological risks, this stage may trigger institutional redesign towards cooperative data infrastructures (Micheli et al. 2020).

Finally, impact assessment incorporates the right to restrict or withdraw AI tools. Where evidence and insights from the collective deliberation phase reveal inequitable outcomes, erosion of consent, or unacceptable risks, communities and managers retain authority to limit systems’ supporting roles, pause automation, or disengage entirely. In practice, this might mean suspending a visitor nudging system over sensitive site access, refusing to renew a contract without data portability, or collectively deciding that certain zones remain outside AI-mediated management altogether. Rather than failure of governance, the right to withdraw is treated as an accountability mechanism, ensuring that the combination of human-centred technical skill, knowledge production, and practical wisdom continues to govern protected area stewardship.

## Conclusion

As technology evolves at a pace that increasingly challenges institutional capacity to assess its consequences, embracing an approach that critically incorporates *techne*, *episteme*, and *phronesis* can help navigate the diverse technical, epistemological, and ethical–political challenges inherent to engaging with AI in sustainability transformations. Reflexive AI governance places human judgement and concerns at the centre, bridging technical designs, normative reflections, and political negotiation. Instead of resolving tensions, it holds them open by supporting collective sensemaking, navigating incommensurable values and ethical dilemmas, while institutionalising questioning of how, why, and whether AI should be used at all. In this sense, it complements the principles advanced in emerging governance frameworks, such as the EU Artificial Intelligence Act and Singapore’s AI governance framework (Camilleri 2024), by extending procedural compliance to sustained democratic deliberation in complex sustainability contexts, asking not only whether a system meets technical standards, but also whether its application is socially legitimate, ecologically proportionate, and culturally appropriate. In this sense, our perspective paper advocates for a shift from efficiency to sufficiency in line with degrowth principles (Kallis and March 2016), emphasising the need and the responsibility to refrain from using AI when its social, ethical, or ecological implications are disproportionate, ensuring that AI remains purposeful, appropriate, and justifiable in its application (van Wynsberghe 2021).

Finally, our perspective paper poses a critical societal question: is there hope for a just and reflexive approach to AI governance that can contribute meaningfully to sustainability transformations? We argue that cultivating critical and democratic governance approaches remains the most credible, and perhaps only, path forward. While such approaches remain aspirational and cannot guarantee success against the increasing concentration of corporate power (Crawford 2021), they offer a foundation for governing AI in ways that are socially legitimate, epistemically just, ecologically grounded, and fundamentally democratic. Against mounting pressure to optimise, automate, and scale, the most consequential governance act may sometimes be to slow down, question, or even refuse the use of AI. Cultivating the institutional capacity and will to do so is as important as any technical capability the system can offer.

## Footnotes

1 https://chatgpt.com
